# Female Micro-Entrepreneurs and Social Networks: Diagnostic Analysis of the Influence of Social-Media Marketing Strategies on Brand Financial Performance

**DOI:** 10.3389/fpsyg.2021.630058

**Published:** 2021-04-12

**Authors:** Ana Isabel Jiménez-Zarco, Jose Antonio Clemente-Almendros, Inés González-González, Jorge Aracil-Jordà

**Affiliations:** ^1^Business Faculty, Open University of Catalonia, Barcelona, Spain; ^2^Marketing Department, Comillas Pontifical University-ICADE, Madrid, Spain; ^3^Business Faculty, International University of La Rioja, Logroño, Spain; ^4^Marconi International University (MIU), Miami, FL, United States; ^5^BLC Group, Madrid, Spain

**Keywords:** women entrepreneurs, micro-SMEs, social media marketing, financial performance, digital transformation

## Abstract

The business world is facing a very complicated situation due to the COVID-19 pandemic. Small- and medium-sized companies (SMEs)—both in Spain and at the global level—are seeing their survival jeopardized by a fall in revenues. This scenario is aggravated in the case of micro-SMEs headed by female entrepreneurs. Accordingly, micro-SMEs, particularly those led by female entrepreneurs, need to reinvent themselves to overcome the current adversities that could lead to the destruction of their businesses and hence their jobs. One of the ways to do this is to take advantage of digital transformation. Therefore, the aim of this paper is to analyze which variables influence the financial results of female-led Spanish micro-SMEs when they carry out social marketing actions. For that purpose, an online survey was designed and analyzed using the “PROCESS” macro. Results show that social media marketing actions have significant effects on financial performance.

## Introduction

Rates of female entrepreneurship in Spain are higher than in the rest of Europe. According to data provided by the 2019 Global Entrepreneurship Monitor (GEM) World Report, for every 10 start-ups in Spain led by men, there are 9 led by women, while the European average stands at six female entrepreneurs for every 10 male entrepreneurs.

Among the reasons for female entrepreneurship is the need to break through the “glass ceiling,” or the importance of achieving a work-life balance (Moreno-Gavara and Jiménez-Zarco, 2019). However, the desire to start up a business is hindered by the fact that the majority of women entrepreneurs—particularly those who set up a micro-SME or are self-employed—face not only economic difficulties but also an ongoing lack of cultural and social support in our country (Mastercard Index of Women Entrepreneurs, 2019). As a result, many of them start their business in the services sector, intensively using the digital environment (Navarro and Martínez, 2012).

The complicated situation facing Spanish women entrepreneurs is being seriously aggravated by the coronavirus disease of 2019 (COVID-19) crisis. The general fall in demand, together with temporary closures and the restrictions imposed on opening hours and maximum capacity, translates into a scenario of uncertainty and risk that leads many to fear for the survival of their business (Spanish Confederation of Small and Medium Enterprises, 2020).

Short-term survival becomes the objective (Caldecott, 2020), and driven by a need to modify their business strategy, many micro-entrepreneurs turn their attention to the digital environment in their area of activity (Joseph et al., 2020). The restrictions imposed on economic activity have also affected the general public: online shopping has become widespread and social networks are playing a leading role as a channel that connects companies and consumers (Laguna et al., 2020). As a result, many companies that are totally new to the digital environment are starting to see social media marketing (SMM) as a simple, inexpensive way to raise awareness and make a name for themselves. The digital environment also lowers the cost of sales, by providing companies with a new channel through which to inform, dialog, and interact with their traditional and new customers (Constantinides, 2014).

The social media landscape has revolutionized marketing by giving smaller companies the ability to run efficient marketing campaigns and create brand awareness more effectively. In the past, only large companies could afford major marketing campaigns, but today, every company can have a presence on social media and use it as a tool in their marketing (Coles, 2017).

Given this situation, the present study has a twofold objective. In the first place, it seeks to analyze the effects of social marketing on the financial performance of micro-entrepreneurs. Second, it aims to identify whether the existing relationship is influenced by other variables relating to business strategy or the personal characteristics of the entrepreneur.

To that end, in the following section, we present the literature review and development of the hypotheses. The section “Data and Variables” describes the databases used in our study as well as the variables of our model. The section “Empirical Methods and Results” explains the empirical strategy applied and presents our findings together with additional robustness tests. Finally, the section “Discussion and Conclusion” discusses the main findings and their implications.

## Literature Review and Hypothesis Development

### Social Media Marketing and Financial Performance

Social networks provide micro-enterprises with a place to carry out their marketing actions. In particular, women-led micro-enterprises have made them the channel through which to inform and interact directly, closely, and interactively with their customers. According to Smith et al. (2015), social media is not only a tool for the exchange of information (Lee et al., 2018), it can also be an influential component of the consumer’s decision-making process.

Currently, the design and implementation of marketing actions in social media is a key focus of companies. Constantinides (2014) defines SMM as companies’ use of social media platforms^1^ to connect them with their audience in order to achieve different strategic goals such as building brand awareness, increasing sales, or driving website traffic. These actions involve publishing content on their social media profiles, listening to and engaging with their followers, analyzing their results, and running social media advertisements.

SMM has become influential in shaping various aspects of consumer behavior such as awareness, attitudes, and purchasing (Hollebeek and Macky, 2019). These channels have even proven effective in strengthening long-term customer relationships (Smith et al., 2015) by delivering emotional and even epistemic value (Wang and Kim, 2017; Prebensen et al., 2018). Social networks deliver value through the intensive use of audiovisual resources (Teng, 2018). Music and video are used to create ephemeral content, which can be made even more vivid and interactive through the use of augmented reality. All this encourages a more natural flow of interaction, but also creates a sense of enjoyment, pleasure and emotion combined with self-expression (Bayer et al., 2016), generating a hedonic experience that can strongly affect the cognitive/affective/emotional lines of social media users (Braun, 2017).

The COVID-19 pandemic has exponentially increased the creative use of social media, by both people and companies. According to Akram and Kumar (2017) social media are online platforms which people use to build social networks or social relations with other people who share similar personal or career interests, activities, backgrounds, or real-life connections. Currently, social distancing and isolation are increasing the social use of these platforms but can also give people time to use them in emotional way. Social life moves to these networks, making them leisure places where people can meet friends and have informal conversations (Block et al., 2020; Ratten, 2020), as well as engaging in long-forgotten hobbies, neglected passions, and unfulfilled dreams (Banerjee and Rai, 2020).

### Hypotheses and Model

In light of the above, we can posit the following hypotheses:

Culnan et al. (2010) and Smith et al. (2015) point out how in the short term there is no direct relationship between the use of social networks and the financial results of companies. Focusing on large North American companies, these studies indicate that increased adoption of social media platforms is not related to differences in financial performance overall. However, they also suggest that in the medium and long terms, there may be a relationship between the use of social media and financial profits. However, studies by Morgan et al. (2009), Shin (2013), and Wang and Kim (2017) show opposite results, finding that SMM is positively associated with financial firm performance for both large firms in industrialized countries and small firms.

According to these ideas, we propose the following:

H1: SMM actions have a direct effect on financial performance.

These effects occur both in the long and short terms. In the long term, SMM is considered to have a positive effect on consumer engagement, resulting in satisfied or loyal customers sharing their positive feelings in interactions with others in their social networks and becoming advocates for the company (Devereux et al., 2020; Nguyen et al., 2020). At a personal level, consumer engagement makes consumers fans of the company, establishing enduring relationships with them, and even encouraging them to proselytize for the company (Hollebeek and Macky, 2019). Moreover, as they become fans of these company’s websites, they tend to be more loyal and committed to the company and are more open to receiving information about it (Lee et al., 2018). At social level, engagement makes consumers develop new connections, becoming advocates for the company through interaction with other consumers and even non-customers on their social media networks (Wang and Kim, 2017).

Along with the increase in customer engagement, in the short term, companies strive to attract attention, even the interest of non-customers. Wang and Kim (2017) demonstrate the capacity of SMM to attract attention, and toward the brand (Wang and Kim, 2017), but this is only achieved if the strategic objectives have previously been set correctly.

Many companies set their social marketing objectives in terms of gaining knowledge about their consumers (Cheung et al., 2020). They are established during the first stages of the relationship between the company and customers, and normally seek awareness and name recognition among customers. They are also aimed at gathering information about the customer in order to establish the buyer’s personality (Aker et al., 2019). On the other hand, properly defined objectives help to establish the financial indicators on which to base performance measurement and the minimum thresholds from which the result obtained is considered successful (Agostino and Sidorova, 2016).

Thus, we propose the following:

H2: There is a direct relationship between the objectives set and financial performance.

The first phase of SMM plan is the definition of the strategic objectives (Adeola et al., 2020). The company defines them according to market opportunities but also its resources and capacities, and for achievement, there are designed and implement a wide range of SMM.

Today’s consumers are more sophisticated, and use social media to search, evaluate, choose, and buy goods and services (Constantinides, 2014). In particular, consumers love for personalized products, but also seek live seeking unique, satisfactory and customized experiences throughout their journey (Bleier et al., 2019). That is the reason because they are keen to get actively involved in the product design, development, and marketing activities (Lin et al., 2018; Zadeh et al., 2019).

Marketers are open to the idea of offering products that can be customized according to the wishes of the final consumer with the aim to increase consumer implication and involvement (Saura et al., 2019). Zadeh et al. (2019) show how companies create, within social platforms, the conditions, means, and tools for consumers to be actively engaged in co-creation processes, both at product design and development, as well as marketing and communication actions aimed to build a strong brand awareness, reputation, or advocacy, among others.

Yadav and Rahman (2017) provide evidence of how the set of SMM actions develops in social networks has important effects on companies’ financial results. Awareness is the first objective of companies’ SMM strategies. The aim is to ensure that the customer gains a certain level of knowledge about the company/brand that makes them continue looking for information and develop a favorable opinion about it (Langaro et al., 2015).

Brand awareness is increased if customers are frequently exposed to company communications (Makrides et al., 2020). Some SMM actions are specially designed to achieve this objective, such as online advertising, storytelling posts, short videos, and other kinds of actions relating to search engine optimization (Tafesse and Wien, 2018). However, social media can also be used to communicate specific information about the company or to generate and exchange customized company content with the company’s existing and potential customers (Bîja and Balaş, 2014). Thus, there are some SMM actions developed on digital platforms that are specially designed to capture leads, or generate loyalty and advocacy, which also have a moderating effect on awareness.

According to the last ideas, we propose that:

H3: The marketing objective influences the effect of SMM actions on financial performance.

Education is commonly perceived to be important for the success of entrepreneurial activity (Chhabra and Goyal, 2019). According to Almarhry et al. (2020), education provides the necessary skills and knowledge for an entrepreneur to manage their daily business requirements, design, implement and assess the action developed, and face all the obstacles and challenges that may arise during their entrepreneurial lives (Shepherd et al., 2020).

Many studies recognize the direct relationship between the training received by the entrepreneur and the financial results obtained. Galvão et al. (2018) show that education improves skills, especially in areas such as selection of opportunities, organization of resources to deal with risks, and development of businesses. The ability to detect business opportunities or assess the resources and capabilities of the company are pointed out in the literature as determinants of companies’ financial success. Mostafiz et al. (2019) point out how the ability of companies to take advantage of certain market opportunities successfully and immediately depends on the ability of entrepreneurs to identify correct opportunities regarding marketing, resources, raw material supplies, and so forth.

This situation is especially relevant when the entrepreneur has received university training in the area of business, communication, or marketing. Note that business education focuses on three different angles of business management: (1) culture/state of mind, (2) behavior, and (3) creating specific situations (Fayolle et al., 2006; Chhabra and Goyal, 2019). Education focused on business management as a matter of culture/state of mind covers the beliefs, values, and attitudes associated with entrepreneurship (i.e., entrepreneurial identity, spirit, or mindset), while education focused on behavior mostly covers skills such as opportunities, making decisions and developing social skills. Finally, education focused on creating specific situations concerns the creation of new firms and entrepreneurial situations. But even when the training received is not specifically related to business and management, Galvão et al. (2020) recognize that higher education improves entrepreneurs’ ability to recognize business opportunities, stimulating their self-esteem, introspection, knowledge, thereby increasing their ability to act and succeed in a complex environments, and enhancing company performance (Chhabra and Goyal, 2019).

Considering the last ideas, we propose that:

H4: There is a direct and positive relationship between education and financial performance.

The success of the organization depends not only on correctly identifying business opportunities but also on other competences and capacities of the entrepreneur such as: (1) the ability to assess whether the company has the necessary resources and capabilities and (2) the ability to design and implement appropriate strategies to take advantage of favorable business opportunities.

The education received by the entrepreneur is essential in these processes because it promotes values and attitudes such as critical spirit, innovation, and creativity, influencing the success of the decisions made (Fayolle et al., 2006). In addition, when decision-making in an uncertain context, the entrepreneur needs personal skills such as self-esteem, introspection, and knowledge (Galvão et al., 2020), which guarantee the company’s continuity and development over time.

Education favors the presence of all the necessary elements in design and strategic planning: knowledge, skills, and attitudes. Decision-making requires knowledge of the context in which to act, hence the entrepreneur needs information about the market, the customer, and technology, in order to identify the threats and opportunities that they face. However, it also requires the necessary skills and attitudes to be able to assess the situation, as well as to be able to define the objectives to be achieved and design the actions to achieve them.

Even in the absence of any of the above elements, education promotes in the entrepreneur the need to continue learning, either informally or informally (Chhabra and Goyal, 2019). Education helps entrepreneurs to put theory intro practice, and it helps them learn to learn; in other words, to pursue and persist in learning, to organize one’s own learning, including through effective management of time and information, both individually and in groups (Halberstadt et al., 2019).

Thus, education is believed to moderate each and every one of the processes that are developed in the company (Almarhry et al., 2020; Shepherd et al., 2020). The definition of objectives, the choice of tools to be used, the strategy that is established, and the choice of indicators through which success will be evaluated, are all influenced by the entrepreneur’s educational level.

Finally, considering these ideas, we propose that:

H5: Education has a moderating effect on the relationship between the marketing actions carried out and financial performance.

H6: Education has a moderating effect on the relationship between the objectives set and financial performance.

H7: Education has a moderating effect on the influence of the objectives on the relationship between marketing actions carried out and financial performance.

**FIGURE 1:**
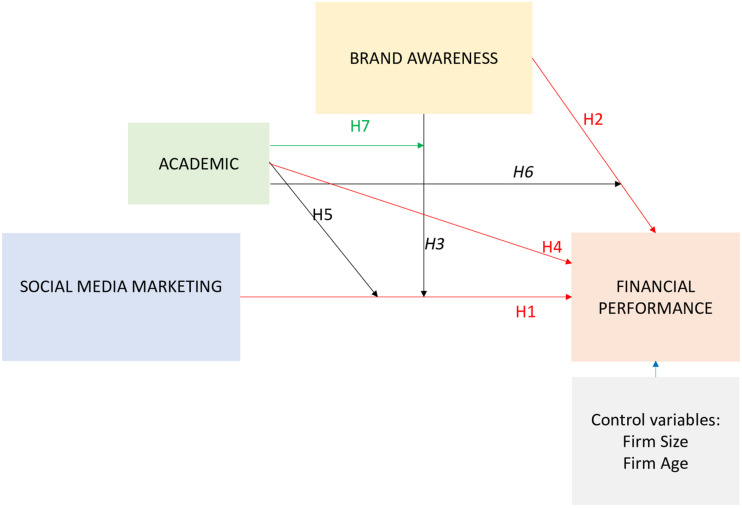
Developed model.

As a summary, Figure 1 shows the model developed.

## Data and Variables

### Data Analyses

We tested our hypotheses by considering a sample of female entrepreneurs from Spain. More specifically, we analyze a group that belongs to the Spanish Federation of Female Directors, Executives, Professionals, and Entrepreneurs (FEDEPE). FEDEPE has been promoting female leadership since 1987, during which time it has been designated as an organization of public benefit. Furthermore, it has held consultative status in the United Nations Economic and Social Council (ECOSOC) since 2012. Its target is women who are unemployed and want to become entrepreneurs or women who are already developing their project and need professional help to continue growing in their business. Our sample belongs to a particular group whose objective is to train participating female entrepreneurs to gain confidence and employability through the development of their own project or improvement of their digital practices. This program aims to improve the visibility of women in the labor market, facilitating female participation and gender equality in business activity. It also seeks to improve their revenues and thus their business performance through the proper use of digital tools.

To obtain the data used to test our hypotheses, we used online surveys, which were carried out between May and June 2020. The participants filled out a survey consisting of 19 questions relating to their background and profile (age, academic background, experience, use of technologies), their goals, type of digital activities developed, and results. We received 127 survey responses, which, after a verification process of the data, where all found to be valid. This represents a response rate of 40.32% of the sample population (315), which is quite satisfactory (Cohen et al., 2008; Chen and Liang, 2011; Pérez-Luño et al., 2018).

We applied standard procedures for survey-based studies. We tested the potential risk of common-method bias with Harman’s one-factor test (Podsakoff et al., 2003). We performed a principal component analysis of all variables in our model. As there was no dominant factor, there is no evidence of common method bias affecting our results (Kerri et al., 2016). In addition, to control for possible non-response bias, we apply the time trend extrapolation test (Armstrong and Overton, 1977) and compare early and late respondents from our survey. This test assumes that late respondents resemble non-respondents. The *t*-test results showed no significant differences in the dependent and independent variables (Camisón and Forés, 2015). Our sample shows similar characteristics in terms of sector of activity, total online sales, percentage of marketing expenses, and experience in social media and digital means (Van Loon et al., 2003).

### Variables

This section describes the variables used to test our hypothesis.

#### Dependent Variable

##### Financial performance (FIPE)

The dependent variable is constructed from the survey data gathered using specific 5-point Likert-scale questions related to the financial performance achieved by the survey respondents. The construct is the result of an exploratory factor analysis (EFA), and the scale is reliable since Cronbach’s alpha is 0.961.

#### Independent Variables

##### Social media marketing (SMM)

This variable measures how intensely the company implements SMM actions to achieve results and improve its financial performance. We add and next average the answers from the 5-point Likert-scale questions related to a list of possible social media actions. Cronbach’s alpha is 0.91, far above the conventional cut-off value of 0.70.

##### Brand awareness (AWARENESS)

This variable assesses the goal of the company. In our case, we focus on the goal: to identify and obtain information about the customer (GOAL_INF). Cronbach’s alpha is 0.78.

##### Academic background (ACADEMIC)

This variable captures the academic background of the female entrepreneur. We distinguish between basic studies, secondary education, university studies, postgraduate studies, and doctoral programs.

#### Control Variables

Our control variables are commonly used in the literature: firm size (SIZE), measured by the number of employees, and firm age (AGE) (Chen et al., 2016; Wang and Kim, 2017; Pérez-Luño et al., 2018).

#### Descriptive Statistics

Descriptive statistics are shown in Table 1. Companies in our sample show an average behavior regarding SMM activities, whereas they depict a clear attitude toward brand awareness. The academic background can be considered a medium-high educational level. The average number of employees is 2.5 with a mean firm age between 5 and 8 years.

**TABLE 1:** Descriptive statistics.

Variables	Obs.	Mean	S.D.	Minimum	Maximum
FIPE	127	0.010	0.959	−1.462	1.761
SMM	127	3.588	1.262	1.000	7.000
AWARENESS	127	5.726	1.343	1.666	7.000
ACADEMIC	127	3.685	0.674	1.000	5.000
SIZE	127	2.535	2.811	0.000	19.000
AGE	127	2.086	1.208	1.000	5.000

*Source: Own elaboration.FIPE, financial performance; SMM, social media marketing; AWARENESS, brand awareness; ACADEMIC, academic background; SIZE, firm age; AGE, firm age.*

As can be seen in Table 2, there is no correlation problem in our sample. Additionally, the variance inflation factor (VIF) values indicate there is no multicollinearity.

**TABLE 2:** Correlation matrix and variance inflation factors*.

	FIPE	SMM	AWARENESS	ACADEMIC	SIZE	AGE
FIPE	1.000					
SMM	0.600 (0.000)	1.000				
AWARENESS	0.268 (0.002)	0.214 (0.015)	1.000			
ACADEMIC	0.054 (0.543)	−0.056 (0.528)	0.054 (0.546)	1.000		
SIZE	−0.030 (0.731	−0.022 (0.800)	0.085 (0.341)	0.156 (0.079)	1.000	
AGE	0.041 (0.645)	−0.017 (0.847)	−0.010 (0.905)	0.082 (0.357)	0.371 (0.000)	1.000
VIF		**1.03**	**1.06**	**1.03**	**1.19**	**1.16**

*Significance levels in brackets. Source: Own elaboration.

## Empirical Methods and Results

We tested our hypotheses by using the moderation approach, with tests of two-way and three-way interactions and bootstrapping to estimate the confidence intervals for the effects (Hayes, 2013).

This approach is limited by the assumptions of normality, and it is particularly recommended when the hypotheses to test include moderating effects and when parametric assumptions are not feasible; for example, in small convenience samples (Russell and Dean, 2000; Hayes, 2013; Pérez-Luño et al., 2018). We use the SPSS “PROCESS” macro to apply the abovementioned approach (Hayes, 2013, 2018a) and to test our hypothesized model depicted in Figure 1.

The “PROCESS” macro is a widely used computational tool for moderation analysis that produces estimates of all the coefficients in the model and generates bootstrap sampling distributions and interval of confidence for the moderation (two-way interaction) and moderated moderation (three-way interaction) through a resampling process with the bias-corrected bootstrapping technique (5,000 bootstrap samples) (Hayes, 2018b; Wang et al., 2018; Tanner and Su, 2019; Wei et al., 2019).

For our three first hypotheses, which do not involve interactions, we use multiple linear regression analysis. The estimated results are statistically robust, as we tested the assumptions regarding regression analysis (Camisón and Forés, 2015). The mean of the residual is zero. In addition, the Durbin-Watson statistic is 2.023, which allows us to assume that the residuals are zero (as the value is between 1.5 and 2.5). The scatterplot of standardized residuals and standardized predicted values confirms their independence, showing homoscedastic variance (Supplementary Figure A-1). The plot of standardized residuals shows that the distribution of the residuals follows the normal probability model (Supplementary Figure A-2).

We need to plot the interactions for a better understanding of the two-way and three-way interactions in our model (Laufs et al., 2016; Pérez-Luño et al., 2018). Following Dawson and Richter (2006), we graph the interactions involved in our hypotheses.

Table 3 presents the coefficients of the direct relations, two-way and three-way interactions. These show that the subsequent addition to the analyzed variables significantly increases the explanatory power of the models, as can be seen by the increase in the *R*-squared values.

**TABLE 3:** Correlation matrix and variance inflation factors*.

Explanatory variables	Model I	Model II	Model III	Model IV	Model V
	**H1 H2 H4**	**H3**	**H5**	**H6**	**H7**

SMM	0.435*** (0.055)	0.065 (0.227)	−0.249 (0.291)	0.440*** (0.055)	1.807 (1.040)
AWARENESS	0.104** (0.052)	−0.110 (0.137)	0.122** (0.051)	−0.071 (0.251)	0.962 (0.492)
ACADEMIC	0.119 (0.102)	0.135 (0.101)	−0.479* (0.269)	−0.155 (0.397)	1.099 (0.753)
SMM * AWARENESS		0.065* (0.038)			−0.361 (0.180)
SMM * ACADEMIC			0.182** (0.076)		−0.467 (0.281)
AWARENESS * ACADEMIC				0.048 (0.068)	−0.283** (0.135)
SMM * AWARENESS * ACADEMIC					0.113** (0.048)
AGE	0.056 (0.060)	0.067 (0.060)	0.054 (0.059)	0.059 (0.060)	0.050 (0.059)
SIZE	−0.023 (0.026)	−0.019 (0.026)	−0.024 (0.025)	−0.024 (0.026)	−0.012 (0.025)
Constant	−2.663*** (0.501)	−1.552* (0.826)	−0.487 (1.033)	−1.692 (1.448)	−5.134* (2.674)
Observations	127	127	127	127	127
*R*-squared (*p*-value)	0.368	0.455 (0.000)	0.421 (0.000)	0.396 (0.000)	0.455 (0.000)

*Standard errors in brackets.Superscript asterisks indicate statistical significance at ***0.01, **0.05, and *0.10 levels. Source: Own elaboration.

In Model I, it can be seen that the SMM actions (SMM) and consumer knowledge together with brand awareness (AWARENESS) show a positive and significant effect, confirming H1 and H2 (coefficients of 0.435, *p* = 0.000 and 0.104, *p* = 0.046, respectively). These results corroborate the idea that the growing adoption of SMM is related to differences in financial performance (Wang and Kim, 2017). It is also confirmed that a clear definition of the company’s goals—more specifically, the mutual awareness and understanding between consumers and company—results in successful financial performance (Agostino and Sidorova, 2016). However, our H4 is not confirmed. Even though the coefficient of ACADEMIC is positive (0.119, *p* = 0.245), it is not significant. While the positive coefficient is in line with the literature (Galvão et al., 2018) showing that as educational level increases, specific skills related to business development improve thereby positively affecting the financial performance of the business; in our sample, it seems that academic background alone does not exert the expected positive effect. This suggests that, when it comes to financial performance, academic background needs the concurrence of additional factors to really exert a positive effect.

**FIGURE 2:**
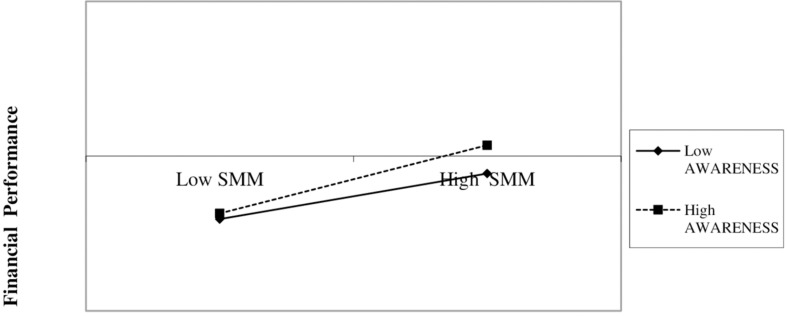
Two-way interaction. SMM * AWARENESS. SSM, social media marketing, how intensely the company implements Social Media Marketing actions; AWARENESS, brand awareness, as the goal of the company.

In Model II, we can see that our H3 is confirmed (coefficient 0.065, *p* = 0.095). The business objective determines the SMM actions developed by the company and thus, the effect on its financial performance (Yadav and Rahman, 2017). For a better understanding of this behavior, we plot in Figure 2 the interaction between SMM and AWARENESS. Figure 2 shows, in line with H1, the positive effect of SMM on financial performance. However, this positive effect is stronger for those companies with greater commitment to improving the company’s and the customers’ knowledge about one another (AWARENESS) (Bîja and Balaş, 2014; Tafesse and Wien, 2018).

**FIGURE 3:**
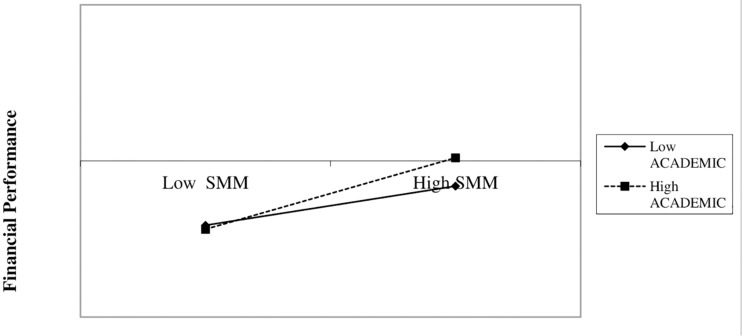
Two-way interaction. SMM * ACADEMIC. SSM, social media marketing, how intensely the company implements Social Media Marketing actions; ACADEMIC, academic background of the female entrepreneur.

Model III confirms H5 (0.182, *p* = 0.018). Academic background moderates all kinds of processes developed in the company (Almarhry et al., 2020; Shepherd et al., 2020). In our model, even though academic background (ACADEMIC) alone is not significant, when it moderates SMM actions, it exerts a positive and significant effect on financial performance. Figure 3 shows that, in line with H1, SMM has a positive influence on financial performance. Nevertheless, said positive effect is stronger when the female entrepreneur has a higher educational level (Fayolle et al., 2006).

**FIGURE 4:**
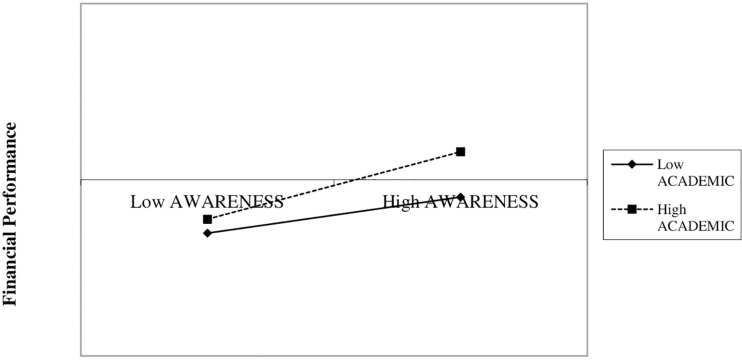
Two-way interaction. AWARENESS * ACADEMIC. AWARENESS, brand awareness, as the goal of the company; ACADEMIC, academic background of the female entrepreneur.

Model IV represents the results of our H6, which cannot be confirmed (0.048, *p* = 0.476). The expected positive moderating effect (Shepherd et al., 2020) of academic background (ACADEMIC) on the influence of better reciprocal knowledge between customers and business (AWARENESS) on financial performance, also stated in H2, exists and is positive but not significant. Figure 4 shows that, in line with H2, companies with higher marketing objectives, aimed at obtaining customer information, obtained better financial performance (Aker et al., 2019; Cheung et al., 2020). This positive effect is stronger when the female entrepreneurs have a higher educational level (Galvão et al., 2018). Nevertheless, this moderation is not significant. Again, as in H4, our results indicate that in order to exert a positive and significant influence on financial performance, academic background needs the concurrence of additional factors.

**FIGURE 5:**
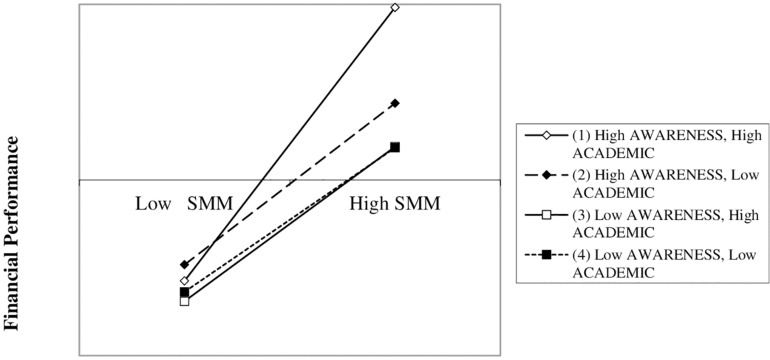
Three-way interaction. SMM * AWARENESS * ACADEMIC. AWARENESS, brand awareness, as the goal of the company; ACADEMIC, academic background of the female entrepreneur; SSM, social media marketing, how intensely the company implements Social Media Marketing actions.

Finally, Model V shows the results of our H7, which is confirmed. The coefficient of the three-way moderation (0.113, *p* = 0.021) indicates that academic background (ACADEMIC) positively moderates the positive effect of AWARENESS on the relationship between SMM and financial performance. Figure 5 helps to gain a better understanding of our three-way interaction (Pérez-Luño, et al., 2018). In line with H3, and regardless of academic background (ACADEMIC), companies with a higher level of SMM activities (Wang and Kim, 2017) and clear business objective—in our case, higher commitment to customer-company reciprocal knowledge (AWARENESS) (Tafesse and Wien, 2018)—show better financial performance. However, said positive effect is stronger when the female entrepreneur has a high educational level, confirming the positive and significant effect of ACADEMIC (Fayolle et al., 2006; Galvão et al., 2018).

## Discussion and Conclusion

According to the results obtained through the analysis carried out by means of the “PROCESS” macro, most of the hypotheses raised at the theoretical level are confirmed. This indicates that the use of SMM actions generates a positive effect on the financial results obtained by the female entrepreneurs.

In particular, these results are observed in the increase in the percentage of sales corresponding to purchases through digital means made by current customers, who had already bought offline before, as well as by new customers, who have got to know the company thanks to the use of the social media.

Thus, on the one hand, the use of social media helps keep the brand in the minds of traditional customers and may even help boost brand recognition. This is fundamental in the current pandemic situation in which companies have been forced to stop their professional activity on a physical level. On the other hand, carrying out such actions also allows companies to establish a first contact with new customer, fostering recognition, and even in the short term securing a first purchase. This is especially relevant because many of these new customers may be from different geographical areas, so not only can the company increase its local sales but it can also reach more distant geographical areas, thus achieving a better regional or national positioning.

Of these two types of customers, it is worth highlighting the figure of the conventional customer, because it is this one that in the very short term has contributed significantly to the increase in sales. This is due to the fact that a substantial portion of the marketing actions carried out by female-led micro-enterprises have been *via* channels such as e-mail and WhatsApp, or female entrepreneurs may have even used their personal Facebook accounts to make themselves known. In addition, a large share of the female entrepreneurs recognize that having a database on their customers is a very useful source of information for the design of commercial actions directed at customers.

An important element to highlight is how the female entrepreneur’s level of education moderates the direct effect of SMM actions on the results obtained, as well as the influence that the objectives established in the strategy have on the latter relationship. It should be noted that a large share of the female entrepreneurs have a high level of education; this means that they have soft skills such as critical thinking and communication skills, which are essential for decision-making, setting objectives to achieve, and the design of actions.

Also, they have other social skills required to identify their target as well as the customer’s needs and expectations. Some of the female entrepreneurs may well have completed specialized studies in the field of business management, reinforcing the abovementioned competences. They would thus have the necessary knowledge for the development of efficient actions in the field of marketing and communications.

These conclusions should be taken with some caution since the study carried out has certain limitations. These include the small size of the sample and the bias toward the profile of the female entrepreneur: relatively young and with a high educational level. This fact encourages us to conduct further research to analyze the existence of differences between different business profiles, in terms of educational level, age, or the sector where they carry out their professional activity.

Furthermore, it would also be interesting to analyze the effects that SMM actions have on other performance indicators other than financial ones, such as those related to organizational processes, or to customer relationships and the construction of a social capital. In fact, the latter are very relevant in companies run by women; in this case, obtaining non-financial results constitutes a differential feature. The use of SMM favors the achievement of this type of results since it encourages the building of relationships between the customer and the brand based on affective and experiential elements.

## Data Availability Statement

The original contributions presented in the study are included in the article/Supplementary Material, further inquiries can be directed to the corresponding author/s.

## Author Contributions

AJ-Z was expert in social media marketing. JC-A has been carried out the methodology. JA-J was expert in business management and in entrepreneurship. IG-G was expert in business management, TIC, and finances. All authors contributed to the article and approved the submitted version.

## Conflict of Interest

JA-J was employed by company BLC Group. The remaining authors declare that the research was conducted in the absence of any commercial or financial relationships that could be construed as a potential conflict of interest.

## Footnotes

1 The major social media platforms are currently Facebook, Instagram, Twitter, LinkedIn, Pinterest, YouTube, and Snapchat.
