# Supplementary figures and images for: Female Micro-Entrepreneurs and Social Networks: Diagnostic Analysis of the Influence of Social-Media Marketing Strategies on Brand Financial Performance
Source: Front Psychol. 2021 Apr 12;12:630058. doi: 10.3389/fpsyg.2021.630058 (PMC8072275; doi:10.3389/fpsyg.2021.630058)

**FIGURE A-1: HOMOCEASTICITY**

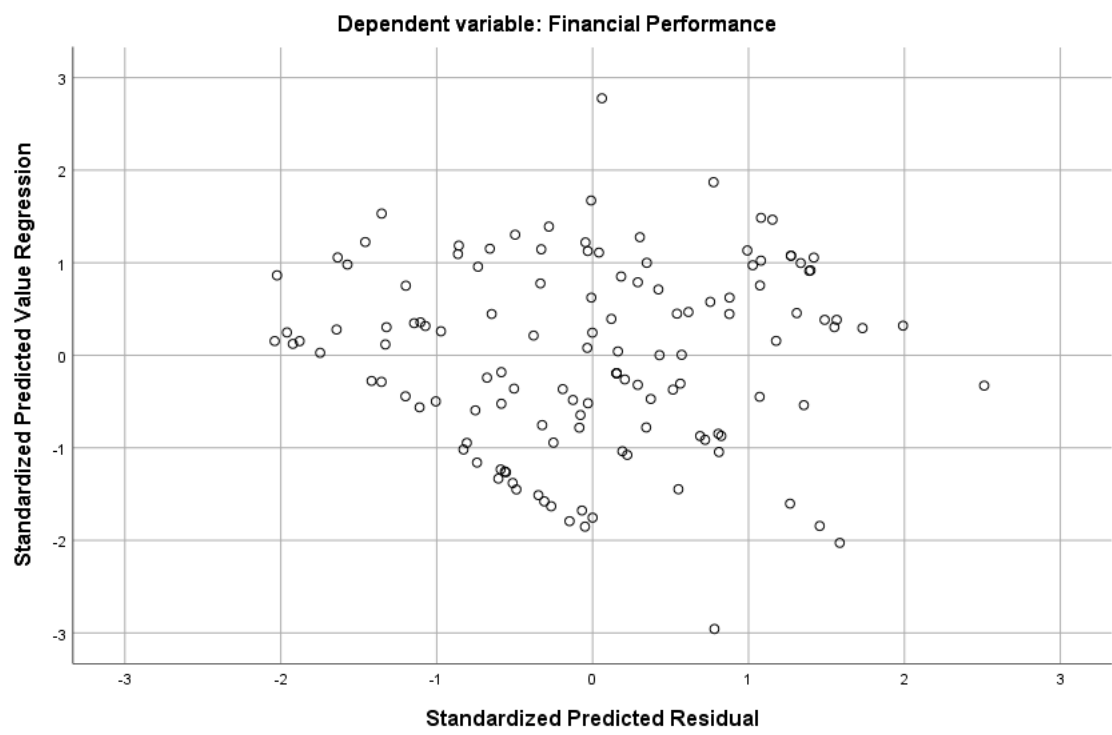

**FIGURE A-2: NORMALITY**

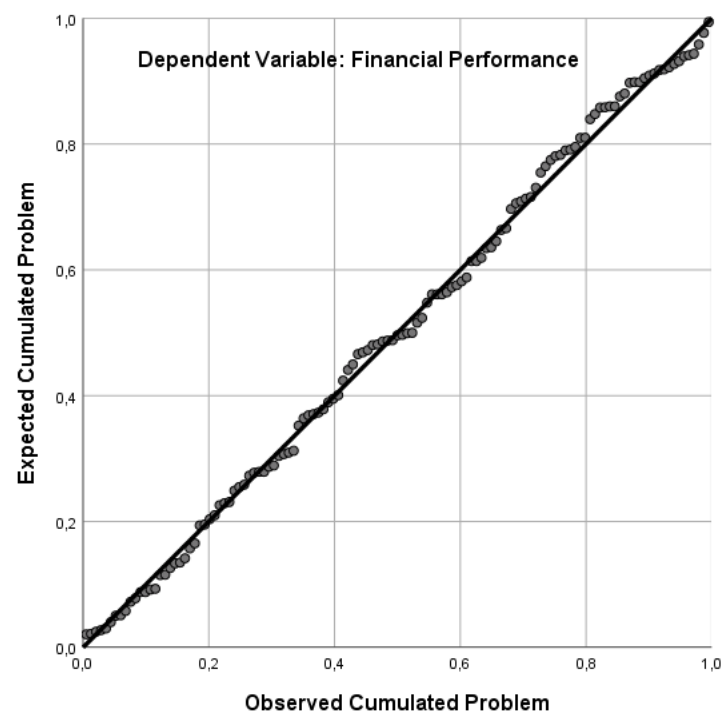

Supplement: Supplementary file 1 [file Image_1.pdf]
